# Submandibular Gland Epithelial-Myoepithelial Carcinoma with Osseous Metastasis: A First Reported Case and Review of the Literature

**DOI:** 10.51894/001c.5042

**Published:** 2016-10-24

**Authors:** Alexander Manteghi, Aaron Centric, Seth Zwillenberg, Corrado Minimo

**Affiliations:** 1 Philadelphia College of Osteopathic Medicine; 2 Albert Einstein Medical Center

**Keywords:** submandibular gland, salivary gland tumor, epithelial-myoepithelial carcinoma

## Abstract

Epithelial-myoepithelial carcinoma (EMC) is a low grade tumor that comprises 1% of all salivary tumors. Local recurrence is not uncommon, but rarely does this tumor demonstrate distant metastasis. We describe a case of a 53-year old female presenting with an asymptomatic, slowly enlarging left submandibular neck mass. Excision of the left submandibular gland (SMG) revealed epithelial-myoepithelial carcinoma with extensive perineural invasion and microscopically positive margins. A subsequent left supraomohyoid neck dissection demonstrated no residual tumor. The patient was stable for one year until a magnetic resonance imaging (MRI) workup for low back pain revealed multiple sclerotic lesions in the iliac crest and lumbar spine, with an iliac crest biopsy demonstrating metastasis. 2.5 year post-operative positron emission tomography-computed tomography (PET-CT) revealed increased [18F]-fluorodeoxyglucose (FDG) avidity in the right iliac crest, pubic symphysis, thoracic and lumbar spine, 9th rib, and sternum concerning for local recurrence and further osseous metastasis. We report the first known case of a submandibular gland EMC with osseous metastasis and highlight the need for prolonged tumor surveillance.

## INTRODUCTION

Epithelial-myoepithelial carcinoma is a rare neoplasm, first described in 1972 by Donath et al.^1^ It has been reported under a variety of names including adenomyoepithelioma, clear cell adenoma, tubular solid adenoma, monomorphic clear cell tumor, glycogen-rich adenoma, glycogen-rich adenocarcinoma, and clear cell carcinoma.^2,3^ It chiefly occurs in the parotid gland, representing about 1% of all salivary gland tumors.^4–6^ It is most common in women (60%) with a peak incidence in the sixth and seventh decades of life.^4–7^ The major salivary glands, and in particular the parotid gland (80%), are the most common sites of occurrence. Submandibular glands comprise 12% of salivary EMCs, respectively.^7^ Clinically, the symptoms are non-specific; a localized, mobile mass is often the only sign. It tends to grow slowly in a bulky, lobulated fashion, demonstrating necrosis and hyalinization of large tumor nodules, and ranges from 2 to 12 cm in size.^5^ Focal invasion occurs often, and single or multiple recurrences complicate the typically protracted postoperative course. Recurrence has been reported to occur from several months to many years after initial excision. While local relapse is not uncommon, distant metastasis is much rarer with only 17 reported cases, predominantly involving the lungs, but also reported in the brain, kidney, skin, and bone. ^6^, ^8–16^ We report the first known case of SMG EMC with distant osseous metastasis, and the third salivary EMC overall with osseous metastasis

## CASE PRESENTATION

**Figure 1.**
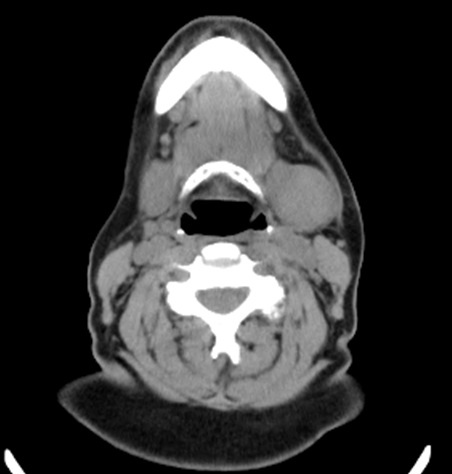
Axial computed tomography without contrast demonstrating a non-homogenous 4.5 x 3.0 x 3.0 cm mass in the left submandibular gland.

**Figure 2.**
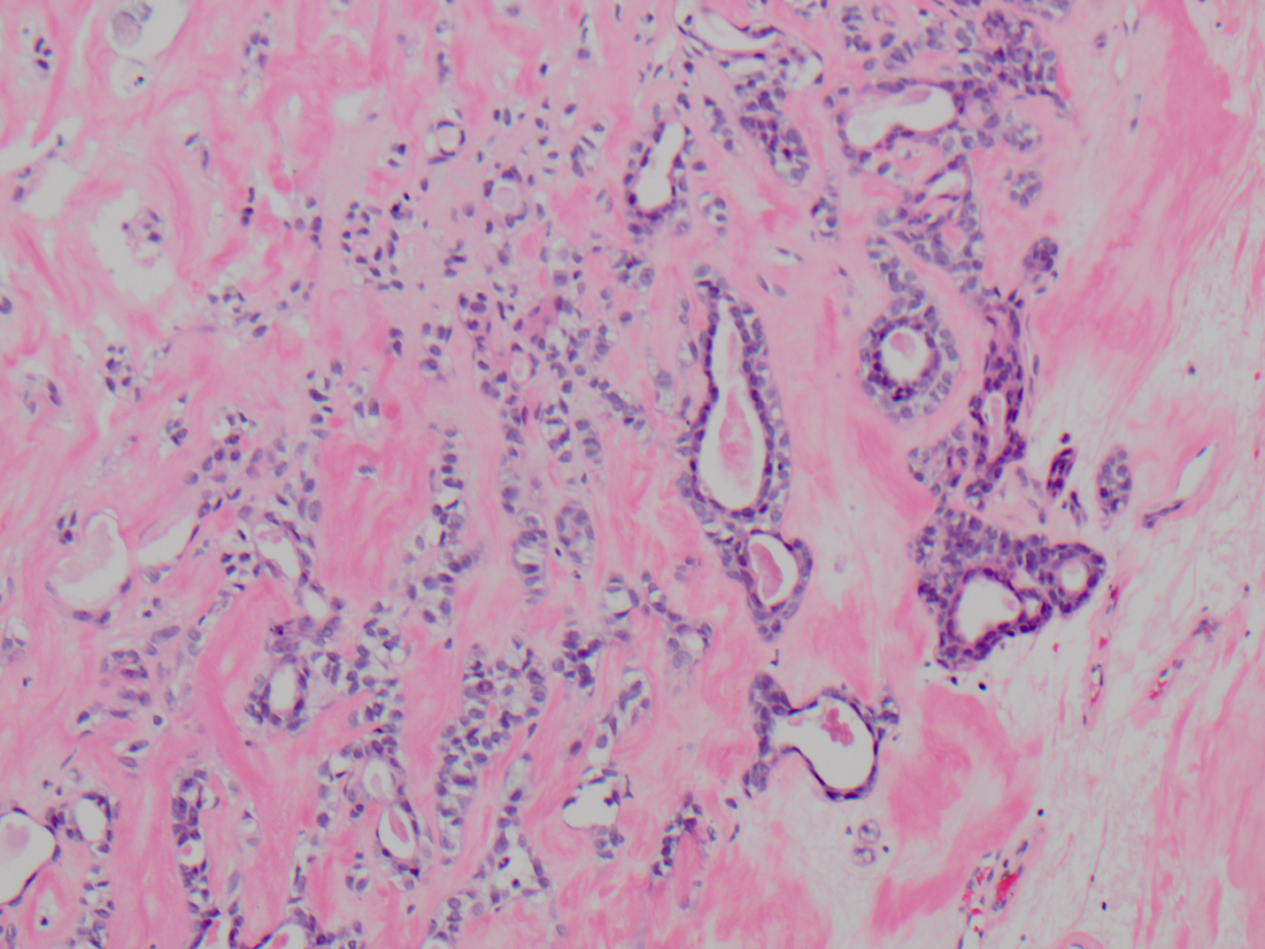
EMC of the SMG. Glandular structures with a luminal layer of eosinophilic epithelial cells are surrounded by larger polygonal myoepithelial cells. Collapsed cords (left) are also present. (H&E, 40x).

A 53-year old female with a 25-pack-year tobacco history and alcohol abuse presented to the otolaryngology clinic with complaint of a ten-year course of slow enlargement of the left submandibular region. A left-sided firm, fixed, and painless submandibular mass was noted on palpation. Non-contrast computed tomography demonstrated a 4.5 cm mass without detectable adenopathy of the neck or surrounding tissues (Figure 1). A non-diagnostic fine needle aspiration of the mass was performed, subsequently followed by SMG excision. Permanent pathology revealed epithelial-myoepithelial carcinoma with a multinodular growth pattern (Figure 2). Immunohistochemical stains for p63 highlighted the respective nuclei and cytoplasm of the outer layer of myoepithelial cells. Calponin and cytokeratin staining was also positive. No lymphovascular invasion was noted; however extensive perineural invasion and positive microscopic margins were identified. Radiation versus further surgery was discussed with the patient. She subsequently underwent a left supraomohyoid neck dissection, including resection of overlying skin. Pathology was benign, and all sectioned lymph nodes were negative for malignancy. The patient declined radiation therapy thereafter, but she continued to have regular surveillance examinations; a yearly surveillance PET-CT was unremarkable. Several months after the PET-CT she later had an MRI workup for low back pain, revealing multiple T2-signal intense sclerotic lesions in several lumbar vertebral bodies and the right iliac crest concerning for possible metastasis. A right iliac crest biopsy demonstrated a small tissue focus with cords of myoepithelial cells embedded in desmoplastic fibroconnective tissue. These cords were morphologically similar to the narrowed cords of the primary submandibular tumor and were also immunohistochemically positive for both cytokeratins and p63. The morphology and the immunophenotype were consistent with metastatic EMC. A subsequent 2.5 year post-operative PET-CT demonstrated increased FDG avidity within the tongue and submental region, concerning for local recurrence, in addition to increased FDG avidity in the right iliac crest, pubic symphysis, thoracic and lumbar spine, right 9^th^ rib, and sternum concerning for further distant osseous metastasis. The patient currently is refusing further intervention.

## DISCUSSION

As the name “epithelial-myoepithelial” suggests, this tumor is composed of a biphasic cell population: an outer layer of clear myoepithelial cells surrounding an inner lining of eosinophilic, cuboidal epithelial cells. The lumen is often occupied by periodic acid-Schiff (PAS)-positive, eosinophilic material. In most cases the cytologic atypia is mild and the mitotic index is low, but there can be exceptions. Although EMC is well differentiated, it often lacks a capsule and shows a multinodular, locally invasive growth pattern, especially prominent through the perineural spaces, as demonstrated in our case. As such, local recurrence is not uncommon.

There are a number of immunoreactive markers. The epithelial cells stain for cytokeratin. Calponin has been reported to be a sensitive marker of myoepithelial differentiation in salivary lesions.^17^ p63 has become a commonly used marker for myoepithelial cells and has been shown to have excellent sensitivity as well.^3^ p63 is not entirely specific, as squamous cell tumors have also demonstrated reactivity.^12^

Since its distinction as a discrete entity, at least 320 EMCs have been described.^3^ The bulk of literature since 1972 has confirmed the tumor to be a low-grade malignancy with documented local recurrence rates ranging from 23% to 80%.^3^ Death related to the primary tumor was found in 40% in one case series.^2^ Interestingly, only 17 cases of distant metastasis have been documented, predominantly involving the lung,^11^ and rarely bone,^2^ brain,^2^ kidney,^1^ and skin.^1^ Our case brings the total to 18 distant metastases, and the first case with osseous metastasis from an SMG EMC.

There is no consensus regarding the optimal treatment of this tumor, largely due to its rarity. Wide surgical excision with a clear margin is the treatment of choice because of the tumor’s tendency to infiltrate locally. If the tumor demonstrates adverse features such as close or positive margins, perineural invasion, lymph node metastasis, intermediate/high grade status, or lymphatic/vascular invasion, then adjuvant radiotherapy is recommended with a consideration for concurrent chemotherapy and radiotherapy (Category 2B Evidence).^18^ The effect of chemotherapy is uncertain. Seethala et al. showed surgical margin status, angiolymphatic invasion, necrosis, and myoepithelial cell anaplasia to be the most significant predictors of survival.^3^

These observations, along with the aggressive nature of our case, lead us to question the assumed low grade malignancy of this tumor. Although the histological features appear to be benign, local recurrence and regional spread occur in an alarming percentage of cases. Distant metastasis, while much rarer, can occur at any point during the clinical course. As an example, the case report of a parotid EMC metastatic to the kidney occurred after 6 local recurrences and 28 years post-parotidectomy.^8^ Long-term close follow-up therefore is necessary, even if the tumor appears to be clinically early stage and completely resected.

## CONCLUSIONS

- We report the first known case of SMG EMC with osseous metastasis.
- EMC is classically a low grade malignancy, but there is a high likelihood of local recurrence. Rarely does this tumor demonstrate distant metastasis.
- The lung appears to be the most common site of distant metastasis, but bone, brain, kidney, and skin also have been implicated, such that no clear metastatic predilection can be identified.
- A margin-free local excision and a long-term close follow-up are necessary, particularly in younger patients.
- Further effort is needed to establish prognostic markers and determine the value of radiotherapy and chemotherapy.

### Conflict of Interest

The authors declare no conflict of interest.
